# Lethal Dermal Sarcoma in Immunosuppressed Patients

**DOI:** 10.1093/oncolo/oyac141

**Published:** 2022-07-22

**Authors:** Marjan Azin, Amir H Ameri, Ruth K Foreman, Victor A Neel, Mayra E Lorenzo, Shadmehr Demehri

**Affiliations:** Cutaneous Biology Research Center, Department of Dermatology, Massachusetts General Hospital and Harvard Medical School, Boston, MA, USA; Center for Cancer Immunology, Center for Cancer Research, Massachusetts General Hospital and Harvard Medical School, Boston, MA, USA; Department of Dermatology, Massachusetts General Hospital and Harvard Medical School, Boston, MA, USA; Cutaneous Biology Research Center, Department of Dermatology, Massachusetts General Hospital and Harvard Medical School, Boston, MA, USA; Center for Cancer Immunology, Center for Cancer Research, Massachusetts General Hospital and Harvard Medical School, Boston, MA, USA; Department of Dermatology, Massachusetts General Hospital and Harvard Medical School, Boston, MA, USA; Department of Pathology, Massachusetts General Hospital and Harvard Medical School, Boston, MA, USA; Department of Dermatology, Massachusetts General Hospital and Harvard Medical School, Boston, MA, USA; Department of Dermatology, Massachusetts General Hospital and Harvard Medical School, Boston, MA, USA; Cutaneous Biology Research Center, Department of Dermatology, Massachusetts General Hospital and Harvard Medical School, Boston, MA, USA; Center for Cancer Immunology, Center for Cancer Research, Massachusetts General Hospital and Harvard Medical School, Boston, MA, USA; Department of Dermatology, Massachusetts General Hospital and Harvard Medical School, Boston, MA, USA

**Keywords:** dermal sarcoma, immunosuppression, organ transplant recipient

## Abstract

Skin cancer is the leading malignancy in immunosuppressed patients, including organ transplant recipients (OTRs), which is increasing in incidence as OTRs live longer. We performed a single-center case series of 4 patients with scalp pleomorphic dermal sarcoma and a history of multiple keratinocyte carcinomas. Outcomes included incidence of dermal sarcoma, dermal sarcoma-related mortality, and histopathologic findings. Out of more than 200 patients followed over a 3-year period in Massachusetts General Hospital High Risk Skin Cancer Clinics, all skin cancer-related deaths (2/2) were due to metastatic dermal sarcoma. Three of 4 patients diagnosed with scalp dermal sarcoma were OTRs and had been on at least one immunosuppressive medication for a median of 9 years. For patients who died from dermal sarcoma, the median time between diagnosis and death was 6 months. Our findings suggest pleomorphic dermal sarcoma contributes to skin cancer-related morbidity and mortality in OTRs.

## Teaser

A single-center case series of four patients with scalp pleomorphic dermal sarcoma and a history of multiple keratinocyte carcinomas is presented. Outcomes included incidence of dermal sarcoma, dermal sarcoma-related mortality, and histopathologic findings.

## Introduction

Skin cancer is a major cause of morbidity and mortality among immunosuppressed patients especially organ transplant recipients (OTRs) and is the leading cause of malignancy in this population.^1-6^ The more immunosuppressed the patient is, the greater the risk of developing skin cancer.^1^ Among the skin cancers, the major contributor to morbidity and mortality is squamous cell carcinoma (SCC), which occurs at a much higher rate in OTRs than that seen in the general population.^1^ Additional malignancies include basal cell carcinoma, melanoma, cutaneous lymphoma, and sarcoma. However, it has been suggested that given the small number of previously reported cases of sarcoma, it is not feasible to determine whether transplantation increases the risk of dermal sarcoma.^1^ Undifferentiated pleomorphic sarcoma demonstrates a varied clinical prognosis, but has been shown to be particularly aggressive in OTRs.^7^

## Report of Cases

### Case 1

**Figure 1.**
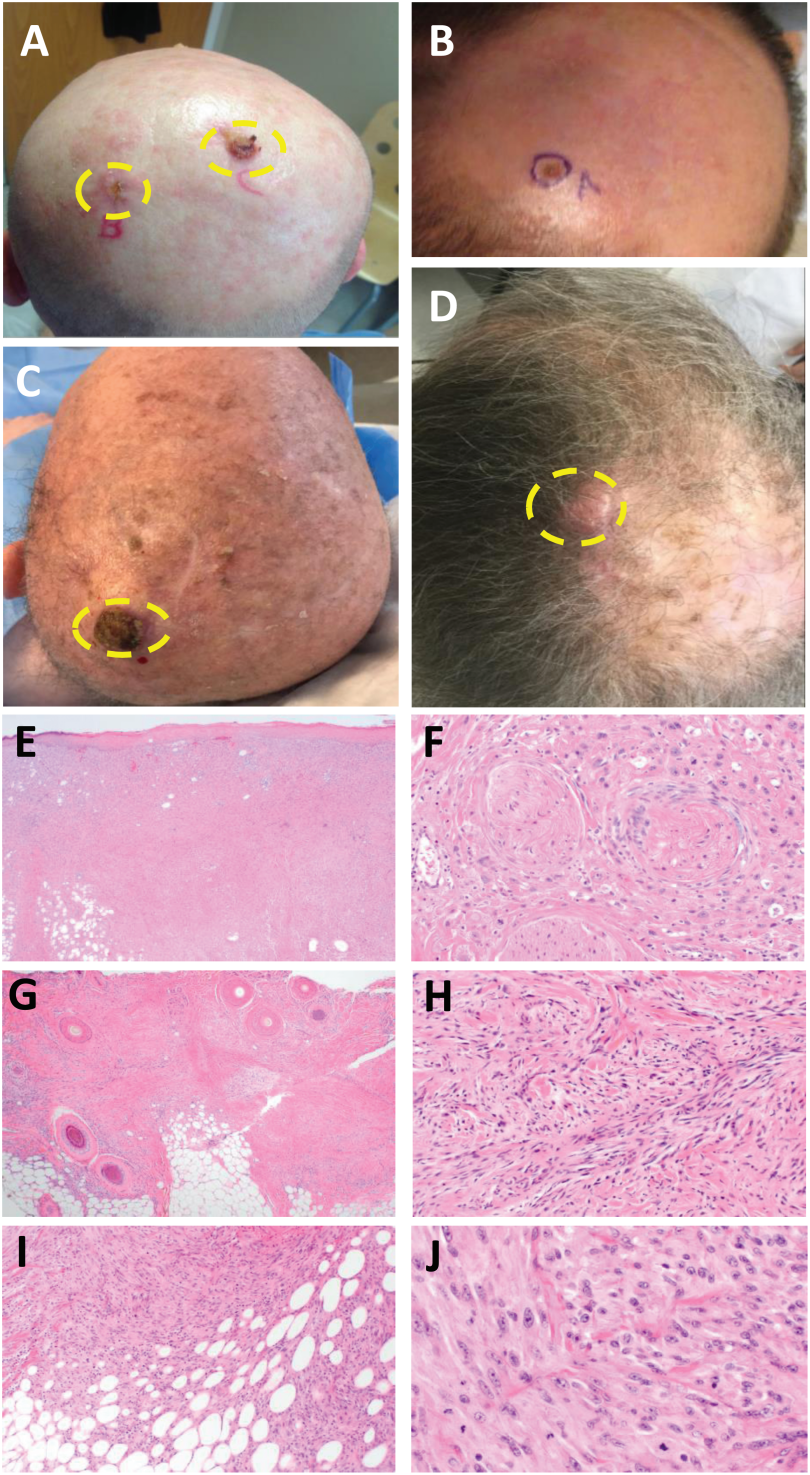
Clinical and pathological examination the patients. (**A**) Clinical image of Patient 1 demonstrates approximately 1.5 cm friable plaque on the medial occiput. To the left of it is an exquisitely tender, ill-defined 1 cm plaque. (**B**) Clinical image of patient 2 showing 0.7 x 0.7 cm crusted papule on the scalp outside former scar. (**C**) Clinical image of patient 3 demonstrating 2 cm hyperkeratotic erythematous nodule on left scalp vertex. (**D**) Clinical image of patient 4 showing smooth, erythematous, 3 cm dermal nodule on the left posterior scalp. (**E**) Hematoxylin and eosin (H&E) stained sections show a spindled cell malignancy with involvement of the dermis and subcutaneous tissue in patient 1. (**F**) Higher magnification images show spindled to ovoid tumor cells with pleomorphism, frequent mitoses and perineural invasion in patient 1. (**G**) H&E stained sections show a spindled cell malignancy with involvement of the dermis and subcutaneous tissue in patient 2. (**H**) High magnification image shows spindled to ovoid tumor cells with pleomorphism and frequent mitoses in patient 2. (**I**, **J**) Medium- and high-power images show spindled to ovoid tumor cells with pleomorphism and frequent mitoses in patient 3.

A 64-year-old male with idiopathic pulmonary fibrosis status post right lung transplant in 2008 and a history of cutaneous SCCs, presented in April 2017 with tender lesions on his scalp of at least 4 months’ duration. He had been on immunosuppressive medication since his transplant, including prednisone, tacrolimus, and mycophenolate mofetil. Physical exam was notable for 2 tender, ulcerated and friable lesions on the posterior scalp (Fig. 1A). Biopsy of the 2 lesions showed (a) spindle cell proliferation consistent with pleomorphic dermal sarcoma and (b) sarcomatoid carcinoma. It was unclear whether these lesions were independent primaries or represented the 2 foci of the same tumor. Wide local excision of the lesions was performed in May 2017, which demonstrated a pleomorphic dermal sarcoma (Fig. 1E and F). Seven months later, the patient progressed to metastatic disease involving the small intestine and he died later that month (Table 1).

**Table 1.** Clinical characteristics of the patients.

Patient/ sex/ age	OTR	Diagnosis	Immuno-suppressants	Years between the start of immuno-suppression and dx	Tumor location	The time between diagnosis and death	Treatment
Pt1/M/64	Yes	Pleomorphic dermal sarcoma	Prednisone Tacrolimus mycophenolate mofetil	9	Scalp	8 months	Wide local excision
Pt2/M/51	Yes	Pleomorphic dermal sarcoma	Prednisone Tacrolimus Rituximab	5	Scalp	3 years	Wide local excision, radiation
Pt3/M/58	Yes	Pleomorphic dermal sarcoma	Prednisone Sirolimus mycophenolate mofetil	22	Scalp	6 months	Wide local excision, radiation
Pt4/M/78	No	Undifferentiated spindle cell sarcoma	N/A	N/A	Scalp	3 months	Wide local excision, radiation

Abbreviations: OTR, organ transplant recipient; Pt, patient; M, male; N/A, not applicable; dx, diagnosis.

### Case 2

A 51-year-old male with cystic fibrosis who received a bilateral lung transplant in 2010 and had been on prednisone, tacrolimus, rituximab, and bortezomib since his transplant presented with a tender papule on his scalp that had appeared several weeks prior to presentation. Biopsy demonstrated pleomorphic dermal sarcoma (Fig. 1G and H), which was treated with Mohs micrographic surgery, adjuvant radiation therapy in early 2015. He subsequently presented in January 2016 with a crusting papule on his scalp that he had noticed several weeks prior (Fig. 1B). Biopsy revealed atypical dermal spindled cell proliferation consistent with residual/recurrent pleomorphic dermal sarcoma. The patient’s new lesion was treated with wide local excision and skin graft. No disease recurrence was detected since the second surgery. The patient died 3 years later due to metastatic colorectal adenocarcinoma (Table 1).

### Case 3

A 58-year-old male with prior kidney transplantation in 1996 and prior excision of an atypical fibroxanthoma in 2012 presented in April 2018 with a 2-cm hyperkeratotic, erythematous nodule on the left vertex of the scalp that had developed rapidly over 3 weeks (Fig. 1C). He has been on sirolimus, mycophenolate mofetil, and prednisone. Excisional biopsy in April 2018 demonstrated a pleomorphic dermal sarcoma with clear margins (Fig. 1I and J). Focal vascular invasion was observed and confirmed with elastin stain. He received adjuvant radiation therapy but died after therapy from complications of immunosuppression (Table 1).

### Case 4

A 78-year-old man with a history of over 50 keratinocyte carcinomas suggesting an immunosenescent state presented in May 2016 for skin cancer screening. He had a history of multiple blistering sunburns and renal cell cancer. He had noted a new growth on his scalp of 2 weeks duration that was not itchy or painful but would bleed with trauma (Fig. 1D). A 4-mm punch biopsy showed a high-grade undifferentiated spindle cell sarcoma extending to tissue edges. Soon after, he developed an additional nodule adjacent to the main mass. Given the satellite lesion, he underwent a staged excision with flap reconstruction and received adjuvant radiation. Pleural biopsy 5 months later demonstrated metastatic sarcoma, to which the patient succumbed 7 months after the initial diagnosis (Table 1).

## Discussion

The diagnosis of 4 patients with dermal sarcoma over 3 years in the Massachusetts General Hospital High Risk Skin Cancer Clinics (MGH HRSCC) suggests that this is a relatively rare tumor type among immunosuppressed patients, especially when compared with other cutaneous malignancies like SCC. Nonetheless, we find that dermal sarcoma is a cause of significant morbidity and mortality in this population. Consistent with our observation, it has been demonstrated that immunosuppression is significantly associated with worse clinical outcomes, poor prognosis and high mortality of undifferentiated dermal sarcoma.^7,8^ In a retrospective study, a higher local recurrence and metastasis of dermal sarcoma have been observed in OTRs compared with immunocompetent patients.^9^ The aggressive nature of dermal sarcomas in our patients is likely secondary to the impaired ability of their immune system to target and destroy the malignant cells. In addition to the patients’ immunosuppressed status, UV exposure may play a contributing role in the initiation of these tumors, especially given their common occurrence on the scalp in this series of 4 Caucasian patients.^1^

Because of the lethality of these tumors, we recommend thorough screening of the high-risk patients for dermal sarcoma as a severe skin cancer in OTRs. These tumors present as subcutaneous nodules; therefore palpation of the underlying subcutaneous tissue should be a part of the screening. Upon detection, these tumors should either receive Mohs micrographic surgery or wide local excision, in addition to possible adjuvant radiation therapy.^10^ Consideration of reducing immunosuppression may be warranted and should be discussed with the transplant team.^1^

The differential diagnosis of dermal sarcoma includes undifferentiated pleomorphic sarcoma, atypical fibroxanthoma, spindle cell melanoma, and spindle cell SCC.^9^ In general, special staining for S100, p63, and cytokeratin help distinguish among these etiologies.^9^ Among these, atypical fibroxanthoma may mimic a low-grade dermal sarcoma with similar clinical features.^8,11^ However, more aggressive and malignant behavior of dermal sarcoma including high number of mitosis and multinucleated giant cells mark the cancers in our patients. This report is limited by a single center retrospective cohort study design, a limited number of patients and the restricted period of patient follow-up. Further research is required to understand the pathogenesis of these tumors in the context of solid-organ transplantation and immunosuppression to prevent the occurrence of these highly lethal tumors.

## Data Availability

The data underlying this article will be shared on reasonable request to the corresponding author.
